# Knowledge of Medical Students and Faculties of a Medical College Towards Human Body and Organ Donation: A Descriptive Cross-sectional Study

**DOI:** 10.31729/jnma.6200

**Published:** 2021-02-28

**Authors:** Poonam Singh, Naveen Phuyal, Sagar Khadka, Minani Gurung

**Affiliations:** 1 Department of Clinical Anatomy, Nepalese Army Institute of Health Sciences, Kathmandu, Nepal; 2 Department of Community Medicine, Nepalese Army Institute of Health Sciences, Kathmandu, Nepal; 3 Nepal Institute of Development Studies, Kathmandu, Nepal

**Keywords:** *body donation*, *knowledge*, *medical students*, *organ donation*

## Abstract

**Introduction:**

The study of clinical anatomy is inseparable from cadaveric dissection. However, scarcity of cadavers is observed all over the world. So, body donation is propounded and is a major source of cadavers worldwide. However, nowadays, there is a scarcity of cadavers for the body dissection in the medical course & also due to the rise in the number of medical institutions in Nepal. This research aimed to find out the knowledge of medical and nursing students at a medical college regarding human body and organ donation.

**Methods:**

A descriptive cross-sectional study was conducted among 400 medical and nursing students in a tertiary care hospital of Kathmandu valley. After obtaining ethical approval, a semistructured questionnaire was used to collect the data. Data were analyzed using Statistical Package for Social Sciences version 20.0.

**Results:**

Most of the respondents, 360 (90%) knew that organs could be donated from living donors as well as cadavers. A majority of the respondents 374 (93.5%) said that bodies could be donated for educational and learning purposes. The eyes were the most commonly donated organ. One hundred seventy five (43.5%) of the respondents were motivated to donate their bodies or organs. Self-motivation followed by motivation through media by celebrities, family members, and faculties were seen among the participants.

**Conclusions:**

We need a proper voluntary body donation act to facilitate medical teaching with the proper motivation of people for this generous gift to further the knowledge and expand the field of medical science.

## INTRODUCTION

The study of clinical anatomy is inseparable from cadaveric dissection. As the number of medical colleges in Nepal has increased over the last few decades, there is a rising demand for cadavers used in medical education and research. In the training of a medical student to become a doctor who can define and address patient issues, explicit knowledge of anatomy plays a fundamental role. As a complex basis for solving problems, the dissected cadaver remains the most effective way of presenting and studying anatomy. However, for dissection, there is an inadequate number of donated cadavers available. Voluntary body donation has become an essential source of cadavers for anatomical study and education. It is defined as an informed and free act of giving one's whole body after death for medical education and research.^1,2^

There is a lack of public awareness and proper information. The donation of human organs is still reluctant due to religious and cultural barriers, myths, and misconceptions concerning organ donation.^3,4^ The time is yet to come to motivate our mind for organ and body donation for the sake of medical science or to save one's life.^5,6^

This research aimed to find out the knowledge of medical and nursing students of a medical college regarding the human body and organ donation.

## METHODS

This is a descriptive cross-sectional study conducted in the medical college, nursing college, and Nepalese Army Institute of Health Sciences tertiary care hospital. After obtaining an ethical approval letter from Institutional Review Committee, data collection was started from October 2020 to November 2020. The respondents were assured of complete anonymity and confidentiality. Email-based consent was used for the study.

Medical students from the College of Medicine, Basic Science and Clinical Faculties, nursing students from the Faculty of Nursing College, and medical officers from a tertiary care hospital were included in the study. Those who gave incomplete answers were excluded.

The sample size was calculated using the following formula,

n = Z^2^ × p × q / e^2^

Where,

n = sample size p= prevalence, 50%^5^
q = 1-p
Z = 1.96 for Confidence Interval of 95%
e = margin of error, 5%

So,

n = (1.96)^2^ × (0.5)(1-0.5)/(0.05)^2^

 = 384

Taking 5% of the non-respondent rate, the total calculated sample size was 403. Participants were selected conveniently. Incomplete answers given by three of the participants were excluded.

A structured questionnaire was used for the study prepared in Google docs; the questionnaire's internal validity was checked by a pretesting and use of Cronbach's alpha. The questionnaire was disseminated to participants through email, Viber, Whatsapp, and Facebook messenger. Data were analyzed using SPSS version 20.0. Appropriate graphical and tabular representation of the data was done.

## RESULTS

Out of 400 respondents, most of the respondents 360 (90%) knew that organs could be donated from living donors as well as cadavers. Nearly, 28 (7%) said that it could only be donated from living donors, and 8 (2%) of the respondents said that organs could only be donated from cadavers. The majority of the respondents 374 (93.5%) said that bodies could be donated for educational and learning purposes.

Out of the total respondents, 391 (97.8%) have donated their organs, and 9 (2.2%) of the respondents have never donated their organs and out of those who had donated their body majority (n=7) were doctors, and two were nurses. All of the respondents who have donated their organs had their eyes donated, and one had donated their kidney.

Less than half 174 (43.5%) of the respondents were motivated to donate their bodies or organs, while 226 (56.5%) were not motivated to donate their bodies or organs. Out of those who were motivated, most of the respondents were self-motivated, followed by motivation through media by celebrities, family members, and faculties.

**Table 1:** Motivators for organ and body donation.

Motivators for Organ/Body Donation	n (%)
Self-motivated by profession	41 (23.5)
Celebrities and Media	43 (24.7)
Family members	33 (18.9)
Faculties	18 (10.3)
Health Workers	18 (10.3)
Other Organ donators	10 (5.7)
Friends	7 (4.0)
Social Workers	4 (2.2)
Total	174 (100)

Nearly half, 180 (45%) of the respondents said that they would donate their organs/after death, and one third, 140 (35%) said that they needed to be convinced for organ donation, while 8 (2%) of the respondents said that they would not donate their organs after death. Out of those who wanted to donate their organs after death, 137 (34.3%) wanted to donate their eyes, 58 (14.5%) wanted to donate any possible body organs, 22 (5.5%) wanted to donate their kidneys, 19 (4.8%) wanted to donate their heart.

**Table 2:** Body organs respondents wanted to donate after death (n = 134).

Organs	n (%)
All possible organs	58 (14.5)
Bone Marrow	1 (0.3)
Eye	137 (34.3)
Heart	19 (4.8)
Kidney	22 (5.5)
Liver	10 (2.5)
Skin	16 (4)
Testis	2 (0.5)
Uterus	1 (0.3)

When asked if the respondents were willing to donate their body after death, 134 (33.35%) said that they were willing, 86 (21.8%) said that they were not willing to donate their body after death, and 181 (44.8%) said maybe they would donate their body after death if convinced. Of those who were willing to donate their body/organs (n=134), a majority 62 (46%) said they would donate it for organ donation. Thirty-five percent (47)o f those willing said both for educational purposes and organ donation. Of those willing 7 (1.8%) said that they would donate it for educational purposes.

Most of the respondents 78 (29.8%) who were unwilling to donate their bodies/organs did not have any specific reasons.

**Figure 3.**
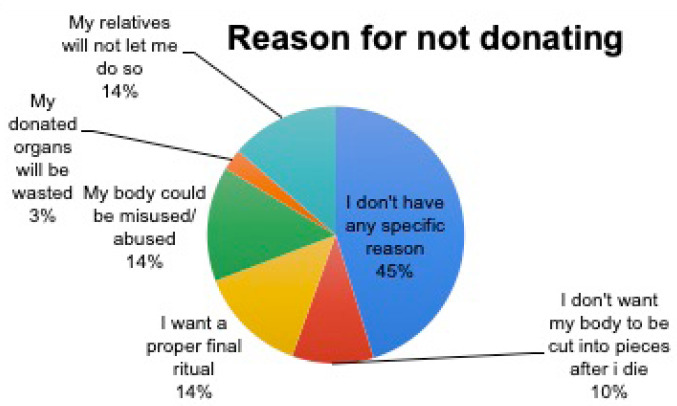
Reasons for not donating body/organs (n = 263).

Three hundred and two (75.5%) respondents wanted to donate their body/organs but have not signed and forms for such purpose. The reasons given by these respondents are shown below in table 3.

**Table 3:** Reasons for willing to donate body/organ but not signed any forms.

Reasons	n (%)
I am interested but not totally decided	95 (23.8)
I am not aware of such form	70 (17.5)
I may change my mind later	29 (7.3)
I will do it later	81 (20.3)
My family has not agreed	10 (2.5)
My relatives have not agreed	1 (0.3)
No benefit	2 (0.5)
Other	14 (3.5)
Total	302 (100)

Out of those respondents who had a positive attitude towards organ/body donation, half (50%) wanted this donation to save others' lives.

**Figure 4.**
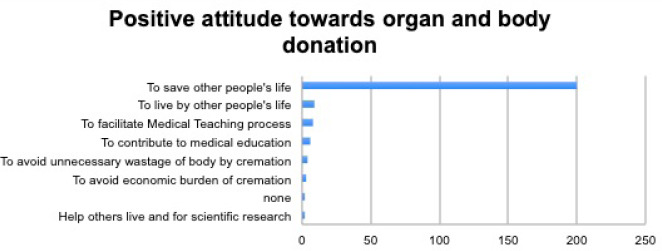
Positive attitude towards organ/body donation.

The median age of the study population was 25 years. Maximum participants 223 (58%) were in the age group of 18-25 years. Sixty percentages of the respondents were female. The majority of the respondents 369 (91.5%) were Hindu by religion, followed by Buddhist 20 (5%). Most of the respondents were doctors 144 (35.8%), followed by medical students 113 (28%), nursing students 73 (18%), nurses 58 (14.5%).

**Figure 5.**
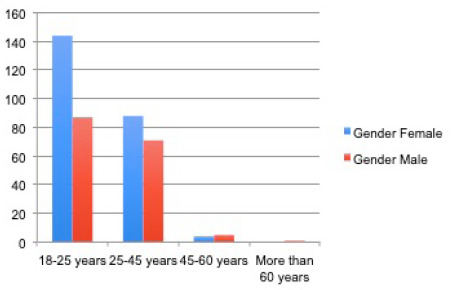
Figure showing Age and Gender.

**Figure 6.**
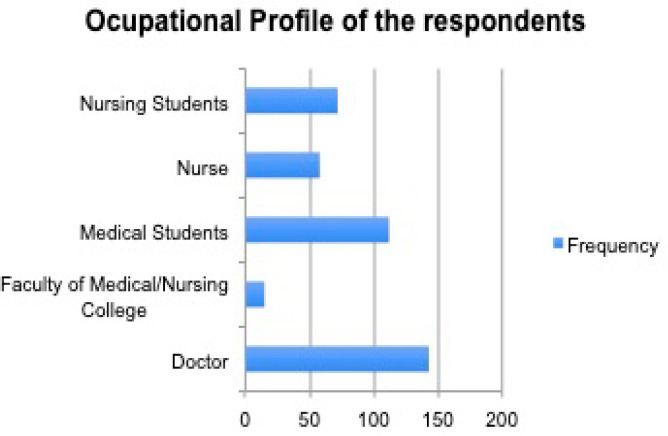
Figure showing the occupational profile of respondents.

More females than males wanted to donate their bodies in this study.

**Table 4:** Cross-tabulation of body donation with gender.

	Gender	Absolutely not	I do not know	Maybe, I need to be fully convinced	Yes without hesitation	Total
Gender	Female	2	33	81	120	236
	Male	9	39	57	59	164
Total	Total	11	72	138	179	400

## DISCUSSION

The majority of our respondents were Hindu by religion, followed by Buddhists. All the religions in the world believe that body and organ donation is a charitable act of giving for teachings in medicine, medical research, and organ transplants. Buddhists believe that body donation is a noble act, which should be done to advance medicine and save lives. Hindu religion also says that donation is an individual's decision but doing good for others is desirable by their religion.^7^

Most of the respondents in our study were doctors and medical students, followed by nursing students and nurses. One study concluded that various factors such as demographic, cultural, attitudinal, and clinical associated with willingness to donate body and organs affect the general public's opinion of body donation. However, these opinions differ in living donations versus cadaveric donations.^8^

Most of our respondents seemed to have a fair knowledge regarding organ and body donation as they responded, saying living as well as cadaveric donors could donate that body/ organ. The majority of our respondents said that bodies/organs could be donated for educational and medical learning purposes. A study conducted in 1992 reported that bodies and organs were donated to help the evolution of medical science and as a gratitude for the medical profession.^9^

More than ninety percent of our respondents have donated their organs, but only a few have donated their bodies. Most of the respondents who donate their organs have done it to save other people's lives, but only a few donate their bodies because of disrespectful behaviors towards cadavers.^10^

More than half of our respondents were not motivated for body donation. Out of those who were motivated, most of them were motivated by celebrities and media, and many were self-motivated through their learning, observation, and clinical practice. Mass media plays a very important role in motivating people for organ/ body donations.^11^ Society has to accept that using body parts is moral and offers a health source for everyone. The greatest impact of motivating people for body and organ donation comes through television, press, and radio, magazines, friends, and families.^7^ Our study suggests that even faculties of medical colleges and health care workers can contribute significantly in motivating individuals for body/organ donation. Most of our respondents wanted their organs donated to save other people's lives, and only a few wanted to donate them for educational purposes, for which motivation is needed substantially. Most of our respondents did not have any substantial reasons for not willing to donate. If this group of people was motivated enough through an effective medium, their numbers could be raised effectively. The decision to donate one's body for learning and research purposes should be based on sound reasoning and convictions.^7^ A similar study also suggested that motivation for cadaveric organ donation is good. However, willingness to donate for teaching purposes is poor, with only 5.66% willing to donate their body for dissection purposes.^3^

Nearly one-third of our respondents wanted to donate their bodies/organs after they were motivated enough. A similar percentage of our respondents did not know about the process of body/organ donation in Nepal. The acts for body donation and organ donation are minimal in Nepal. A study conducted in India said that 99% of the respondents did not know about body donation.^2^ The Anatomy Act in India enacted by various states supplies unclaimed bodies to medical institutions for dissection and medical learning purposes.^7^ Medical institutions of Nepal also acquire cadavers from unclaimed bodies for medical learning purposes, but the supply seems to be minimal with increasing demand. The total cadaver: student ratio ranges from 1:10 to 1:25, which clearly shows that many medical students are not having proper opportunities for dissection, which may hamper their anatomical and clinical skills.^2^

More females than males in our study wanted their organs/body donated, which tells us that more males have to be motivated for body/organ donation. Compared to other countries, the number of respondents wanting to donate their bodies after death is less in Nepal. Other studies suggested that the numbers were higher in India (67.33%), nearly 70% in Turkey, 60% in Spain, and 61% in China.^11-13^

## CONCLUSIONS

Teaching and learning of anatomy depend solely on cadaveric dissection. The saving of human lives depends upon voluntary organ donation, which is a precious gift for mankind. Unclaimed cadavers remain a principal teaching tool for medical educators, and voluntary body donation has not evolved properly in Nepal yet. We need a proper voluntary body donation act to facilitate medical teaching with the proper motivation of people for this generous gift to further the knowledge and expand the field of medical science.
